# External validation of three risk prediction models for deep vein thrombosis in patients with acute stroke: a single-center cohort study

**DOI:** 10.3389/fcvm.2026.1753784

**Published:** 2026-04-10

**Authors:** Lina Fu, Kairu Feng, Chunyan Cui, Lanjun Li, Bing Zou, Nan Liu

**Affiliations:** 1 Institute of Environment and Health, South China Hospital, Medical School, Shenzhen University, Shenzhen, China; 2 Institute of Chronic Disease Risks Assessment, School of Nursing and Health, Henan University, Kaifeng, China

**Keywords:** acute stroke, deep vein thrombosis, systematic review, external validation, risk prediction model

## Abstract

**Background:**

To evaluate the predictive performance of three previously established risk models for deep vein thrombosis (DVT) in patients with acute stroke and to support model selection in clinical practice.

**Methods:**

In this single-center spatial validation cohort study, patients diagnosed with acute stroke and admitted to the Stroke Center of South China Hospital of Shenzhen University between January 2023 and January 2025 were consecutively enrolled. Three DVT prediction models, previously identified by a systematic review conducted by our research team, were selected for external validation. Model performance was assessed by the area under the receiver operating characteristic curve (AUC), Brier score, bootstrap calibration curves, and decision curve analysis (DCA).

**Results:**

A total of 1,270 patients with acute stroke were included, among whom 217 developed DVT, yielding an incidence of 17.08%. The AUCs were 0.699, 0.804, and 0.753 for the Shen Xiaofang, Lu Qiufang, and Xi Pan models, respectively. The Lu Qiufang model achieved the highest positive predictive value (48.4%), specificity (84.9%), accuracy (82.1%), and Youden index (0.536). All three models had negative predictive values exceeding 90%. The Brier scores were 0.182, 0.154, and 0.245. Calibration curves indicated that the Lu Qiufang model demonstrated the best goodness-of-fit, whereas the Shen Xiaofang and Xi Pan models exhibited systematic bias in certain risk intervals. DCA curves showed that the Lu Qiufang model provided greater net benefit within the threshold probability range of approximately 0.20–0.70, indicating superior clinical decision value.

**Conclusion:**

All three DVT risk prediction models demonstrated acceptable predictive performance in patients with acute stroke. Among them, the Lu Qiufang model showed comparatively superior discrimination, calibration, and clinical net benefit. However, given the single-center design of this study, further multicenter and cross-regional validation studies are warranted to confirm model transportability and generalizability across diverse healthcare settings.

## 1 Introduction

Venous thromboembolism (VTE), comprising primarily deep vein thrombosis (DVT) and pulmonary embolism (PE), is a common complication in patients with stroke (1, 2). DVT is characterized by abnormal blood coagulation within deep veins, leading to impaired venous return (3). This complication typically emerges as early as the second day after stroke onset and peaks between 2 and 7 days (4). Without timely and effective intervention, the risk of DVT increases markedly in patients with acute stroke. Within 2 weeks of stroke onset, the incidence of DVT may reach as high as 30%–80% (5). Therefore, DVT is recognized as an independent risk factor for increased in-hospital mortality and worse long-term outcomes in patients with stroke (5). Therefore, early identification of DVT risk in patients with acute stroke is essential for optimizing patient management and improving clinical outcomes (6).

Currently, numerous DVT risk prediction models have been developed for patients with acute stroke. Based on a prior systematic review, our research team identified three models with relatively good predictive performance, accessible clinical variables, and strong applicability: the Shen Xiaofang model (7), the Lu Qiufang model (8), and the Xi Pan model (9). In their internal validations, all three models demonstrated good discriminative ability, with areas under the receiver operating characteristic (ROC) curve (AUCs) of 0.851, 0.893, and 0.811, respectively. However, these three models we have mentioned have not yet been evaluated for the predictive performance in target populations from other regions or hospitals, and their value in broader clinical implementation remains unclear (10, 11).

External validation is a critical step in assessing the transportability and generalizability of clinical risk prediction models. According to the data source, external validation can be classified into temporal, spatial, and domain validation (12, 13). Among them, spatial validation refers to the use of data from other centers or countries to assess a model's performance, and it is generally considered more robust than temporal validation (14, 15). Therefore, this study adopted spatial validation as a form of external validation. Three previously developed models were applied to an independent dataset of patients with acute stroke to predict the risk of DVT. The aim was to evaluate their predictive performance and provide empirical evidence to support the clinical implementation of these models.

## 2 Methods

### 2.1 Selection of risk prediction models

A systematic literature search was conducted in PubMed, Embase, Web of Science, the Cochrane Library, China National Knowledge Infrastructure (CNKI), Wanfang Database, VIP, and SinoMed from database inception to December 1, 2024. The full electronic search strategy for PubMed is provided in Supplementary Table S1. Before this external validation, our research team conducted a systematic review of DVT prediction models in patients with stroke in accordance with the TRIPOD-SRMA reporting guideline, and the protocol was registered in PROSPERO (CRD42024603132). The main search terms included “Acute Stroke,” “Stroke,” “Cerebral Stroke,” “Venous Thromboembolism,” “Deep Vein Thrombosis,” “Risk Prediction Model,” “Risk Score,” “Risk Factors,” “Predictors,” “Nomogram,” and “Model.” Duplicate records were identified and removed using EndNote 21 software. Two researchers trained in evidence-based medicine independently screened retrieved studies and extracted relevant data according to predefined inclusion and exclusion criteria. In total, 3,804 records were identified up to December 1, 2024, and 23 studies were ultimately included after deduplication and screening according to prespecified eligibility criteria. Eligibility criteria were developed based on the PICOTS framework and the CHARMS checklist; studies were eligible if they developed and/or validated multivariable (≥2 predictors) prediction models for DVT in imaging-confirmed stroke populations. Reviews, case reports, duplicate publications, and studies without accessible full text or key model information were excluded; full eligibility criteria are provided in the Supplementary Material. Subsequently, the risk of bias and applicability of included prediction models were independently assessed by two researchers using the Prediction Model Risk of Bias Assessment Tool (PROBAST) (16–18). PROBAST assessments were used to inform considerations of applicability and reporting completeness, but were not the sole criterion for excluding a model from consideration. The detailed PROBAST assessment results are shown in Supplementary Table S4. Based on the results of the systematic review, models eligible for external validation were further screened according to predefined selection criteria. Ultimately, three prediction models were selected for external validation based on the following criteria: (1) the predicted outcome was DVT (rather than a composite outcome such as VTE); (2) complete model specifications were available, including regression coefficients, enabling calculation of individual predicted probabilities; (3) the model was developed for a general acute stroke population rather than a specific subgroup; and (4) the predictors included in the model were routinely obtainable clinical variables during hospitalization. Basic characteristics of the three selected models are summarized in Table 1. A comprehensive overview of all identified prediction models from the systematic review is provided in Supplementary Table S3. Variable coding schemes and regression coefficients of the three selected models are shown in Supplementary Table S5.

**Table 1:** Basic characteristics of the three DVT risk prediction models.

Model	AUC	Study population	Model development method	Study design	Predictors
Shen Xiaofang	0.851	Acute stroke patients	Logistic regression	Retrospective	Age, diabetes mellitus, dyslipidemia, Padua score, D-dimer, muscle strength
Lu Qiufang	0.893	Acute stroke patients	Logistic regression	Prospective	Age, use of dehydrating agents, degree of hemiplegia, state of consciousness
Xi Pan	0.811	Acute stroke patients	Logistic regression	Prospective	Age, sex, stroke type, malignant tumor, lower limb muscle strength, serum albumin, D-dimer

AUC, area under the receiver operating characteristic curve.

### 2.2 Study population

This independent spatial external validation cohort study included 1,270 consecutive patients with acute stroke who were admitted to the Stroke Center of South China Hospital of Shenzhen University between January 2023 and January 2025 and met the predefined inclusion and exclusion criteria. The inclusion criteria were as follows: (1) age ≥18 years; (2) diagnosis consistent with established criteria for stroke (19); (3) first-ever stroke in the acute phase (disease duration <14 days); and (4) no confirmed diagnosis of DVT or PE at admission. The exclusion criteria were as follows: (1) severe coagulopathy or hematologic disorders; (2) receipt of anticoagulant or antiplatelet therapy within 3 months before admission; and (3) length of hospital stay ≤24 h. The study was approved by the Medical Ethics Committee of South China Hospital of Shenzhen University (No. HNLS20241204003-A). All personal information and medical records of the participants were kept strictly confidential. In addition, the study population and inclusion and exclusion criteria in the present validation cohort were aligned with those of the original model development studies. Predictor variables were defined and coded strictly according to the original model specifications, ensuring methodological compatibility for external validation.

### 2.3 Outcome measure

The primary outcome measure in this study was DVT. The diagnosis was made according to the guidelines for the diagnosis and treatment of DVT (20). To clarify the outcome ascertainment process, all patients underwent lower extremity venous ultrasonography within 24 h of admission and again on days 7 and 14 after stroke onset. Additional ultrasonography was performed during hospitalization if clinical signs suggestive of DVT occurred. Ultrasound examinations were performed and independently interpreted by certified sonographers. Treating clinicians and sonographers were not involved in the calculation of the prediction model risk scores. Model-related variables were uniformly extracted from the electronic medical record system by the research team, and model calculation and statistical analyses were conducted by independent investigators. DVT events were restricted to those occurring during hospitalization.

### 2.4 Data sources and collection process

Before the start of the study, the research team developed a structured data collection form based on the predictive factors included in the three selected models and provided standardized training to all data collectors. The training covered diagnostic criteria for acute stroke, diagnostic methods for DVT, and the timing and assessment standards for each predictive factor. A pilot data collection was conducted in 30 patients with acute stroke to optimize the data collection workflow and refine the questionnaire design. After finalizing the procedures, formal data collection was initiated. Data on the predictive factors were extracted by the research team from the hospital's electronic medical record system and paper-based medical charts.

### 2.5 Statistical analysis

The database was established using Microsoft Excel 2021 (Microsoft Corporation, USA) by two independent researchers. Data entry was verified through a double-check procedure to ensure accuracy. Statistical analyses were carried out in R (version 4.3.3). The distribution of continuous variables was examined with the Shapiro–Wilk test. Data conforming to normality were expressed as mean ± standard deviation $\left(\bar{x}\pm s\right)$, and differences between groups were analyzed with independent sample *t*-tests. For non-normally distributed data, values were presented as median (*P*_25_, *P*_75_), and group comparisons were performed using the Mann–Whitney *U* test. Categorical variables were reported as frequencies and percentages [*n* (%)], and the group differences were assessed using the *χ*^2^ test or Fisher's exact test. All statistical tests were two-sided, and *P* < 0.05 was considered statistically significant (*α* = 0.05). External validation of the three prediction models was conducted through the following procedures. The area under the ROC curve (AUC) with its 95% confidence interval (CI) was estimated using the pROC package. Sensitivity, specificity, accuracy, positive predictive value (PPV), negative predictive value (NPV), and the Youden index were determined according to the optimal cutoff identified from the ROC curve. Classification performance was assessed using confusion matrices generated with the caret package, and PPV and NPV were further computed using the epiDisplay package. Model calibration was evaluated through bootstrap-based calibration curves generated with the rms package. Clinical utility was examined using decision curve analysis (DCA) with the rmda package, which estimated net benefit across different threshold probabilities. The Brier score was applied to quantify the overall prediction error of each model. This study was conducted following the Transparent Reporting of a Multivariable Prediction Model for Individual Prognosis or Diagnosis: The TRIPOD Statement (12), and the recommendations of Evaluation of Clinical Prediction Models (Part 2): How to Undertake an External Validation Study (15).

## 3 Results

### 3.1 Inclusion of study subjects

**Figure 1:**
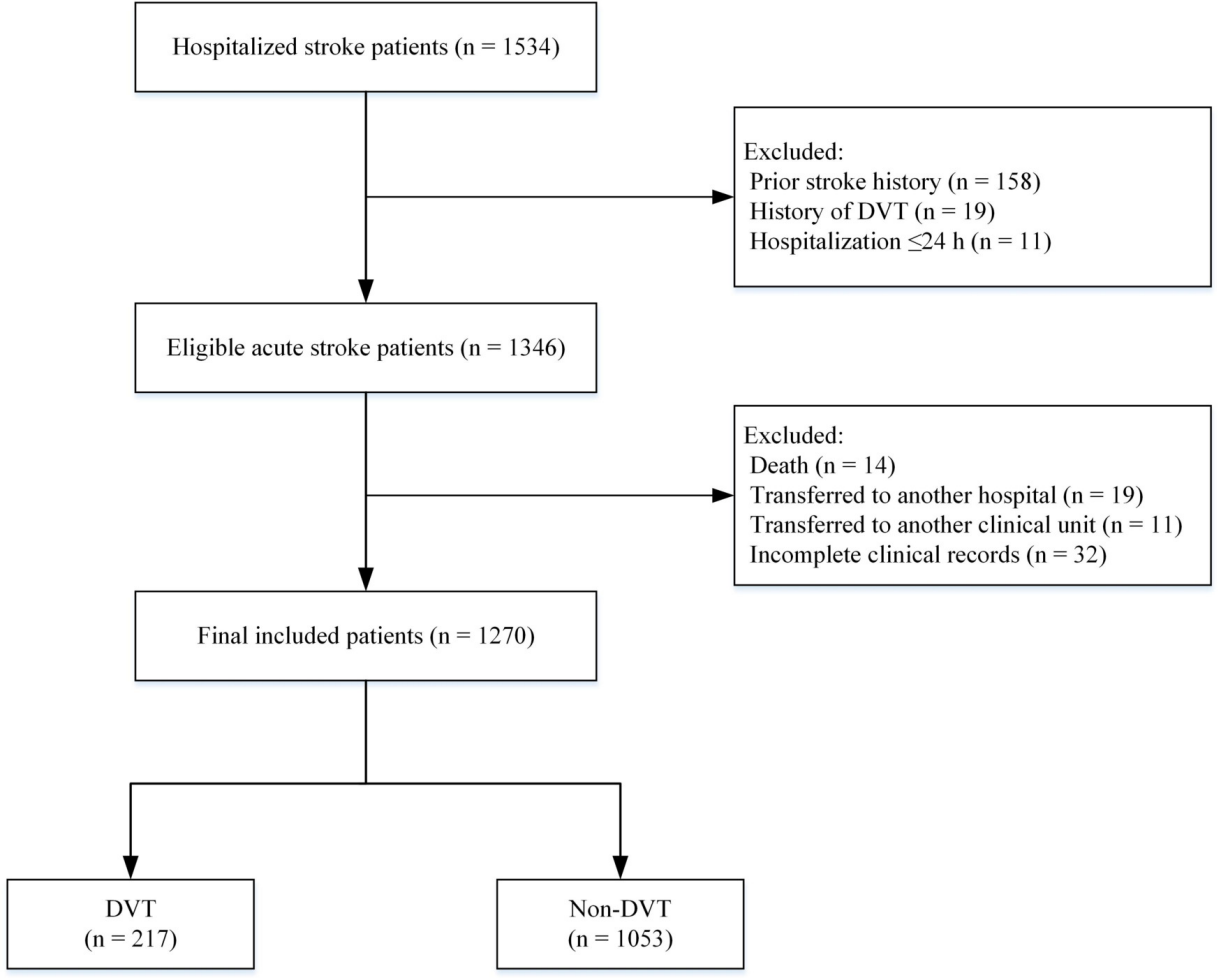
Flowchart of inclusion and exclusion of study participants.

Patients hospitalized in the Stroke Center at South China Hospital of Shenzhen University were selected as the study population for this study. Of the 1,534 patients initially screened, a stepwise screening procedure was conducted according to inclusion and exclusion criteria in Section 2.1. Firstly, 188 patients without meeting the inclusion criteria were excluded, and 1,346 patients were included with acute stroke for further evaluation. Subsequently, 76 patients were excluded due to death, transfer to other hospitals or departments, or missing key examination data, etc. Ultimately, a total of 1,270 patients were included in the external validation analysis. The flow of patient inclusion and exclusion is presented in Figure 1.

### 3.2 Comparison of baseline characteristics

A total of 1,270 patients diagnosed with acute stroke were enrolled and stratified into DVT and non-DVT groups based on the presence or absence of thrombosis. Marked differences in baseline characteristics were identified between the two groups. Relative to the non-DVT group, patients with DVT were older, exhibited higher Padua scores and D-dimer levels, and had reduced lower limb muscle strength (all *P* < 0.001). The prevalence of diabetes mellitus was also higher in the DVT group (*P* = 0.002). In addition, impaired state of consciousness, more severe hemiplegia, use of dehydrating agents, hemorrhagic stroke, serum albumin <40 g/L, malignant tumor, and lower limb muscle strength < grade 3 were all more common in the DVT group (all *P* < 0.001), indicating a higher risk of DVT in these patients. No significant differences were found between the two groups in sex or dyslipidemia (*P* > 0.05). A detailed comparison of baseline characteristics is presented in Table 2.

**Table 2:** Baseline characteristics of patients with and without DVT.

Variables	DVT (*n* = 217)	Non-DVT (*n* = 1,053)	Test value	*P*
Age (years)	63.99 ± 13.28	58.64 ± 11.95	−5.490^a^	<0.001
Padua score	3.00 (2.00–4.00)	2.00 (1.00–3.00)	−7.408^c^	<0.001
D-dimer (mg/L)	0.68 (0.33–1.42)	0.27 (0.22–0.50)	−11.591^c^	<0.001
Muscle strength	3.00 (1.00–4.00)	5.00 (4.00–5.00)	15.098^c^	<0.001
Diabetes mellitus			9.995^b^	0.002
No	139 (64.1%)	785 (74.5%)		
Yes	78 (35.9%)	268 (25.5%)		
Dyslipidemia			0.060^b^	0.807
No	137 (63.1%)	674 (64.0%)		
Yes	80 (36.9%)	379 (36.0%)		
State of consciousness			129.482^b^	<0.001
Alert	121 (55.8%)	906 (86.0%)		
Drowsy	52 (24.0%)	106 (10.1%)		
Stupor	15 (6.9%)	24 (2.3%)		
Coma	29 (13.4%)	17 (1.6%)		
Degree of hemiplegia			292.748^b^	<0.001
Normal	53 (24.4%)	802 (76.2%)		
Mild hemiplegia	37 (17.1%)	128 (12.2%)		
Partial hemiplegia	77 (35.5%)	97 (9.2%)		
Complete hemiplegia	50 (23.0%)	26 (2.5%)		
Use of dehydrating agents			61.211^b^	<0.001
No	82 (37.8%)	697 (66.2%)		
Yes	135 (62.2%)	356 (33.8%)		
Sex			0.117^b^	0.732
Male	135 (62.2%)	642 (61.0%)		
Female	82 (37.8%)	411 (39.0%)		
Stroke type			22.731^b^	<0.001
Ischemic stroke	106 (48.8%)	695 (66.0%)		
Hemorrhagic stroke	111 (51.2%)	358 (34.0%)		
Lower limb muscle strength			212.339^b^	<0.001
≥3	111 (51.2%)	957 (90.9%)		
<3	106 (48.8%)	96 (9.1%)		
Malignant tumor			10.501^b^	0.001
No	184 (84.8%)	967 (91.8%)		
Yes	33 (15.2%)	86 (8.2%)		
Serum albumin (g/L)			—^d^	<0.001
<40	124 (57.1%)	380 (36.1%)		
40–55	87 (40.1%)	657 (62.4%)		
>55	6 (2.8%)	16 (1.5%)		

^a^ *t*-test.

^b^ *χ*^2^.

^c^ Mann–Whitney *U* test.

^d^ Fisher's exact test.

### 3.3 Discrimination and classification performance of the prediction models

**Figure 2:**
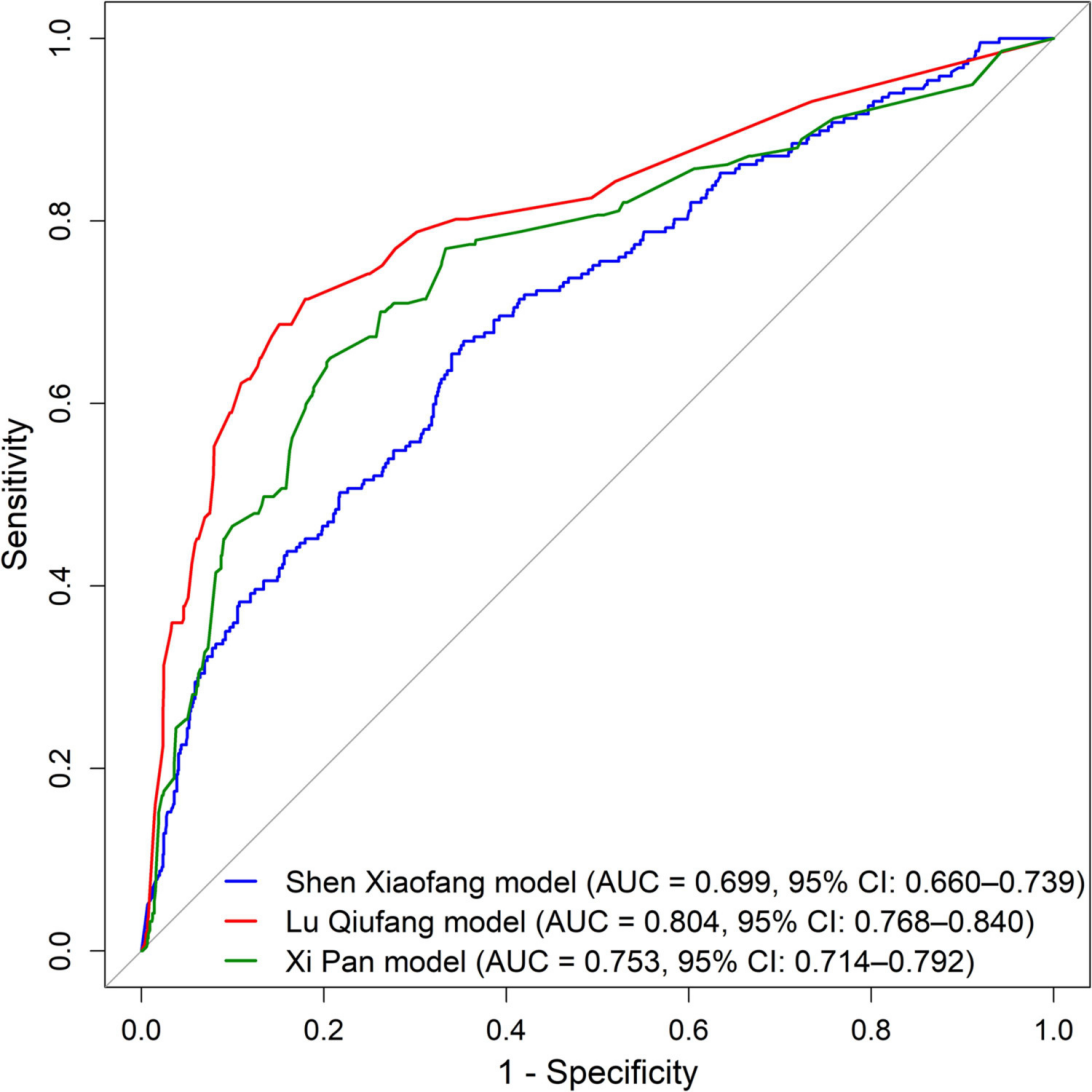
Comparison of ROC curves among three DVT risk prediction models.

The AUC was used to evaluate the discriminatory ability of the three prediction models in the external validation set. The Shen Xiaofang model achieved an AUC of 0.699 (95% CI: 0.660–0.739), the Lu Qiufang model an AUC of 0.804 (95% CI: 0.768–0.840), and the Xi Pan model an AUC of 0.753 (95% CI: 0.714–0.792). All three models showed *P* values <0.001 by the DeLong method, indicating statistically significant discriminatory ability in identifying the risk of DVT. ROC curves of the three models were shown in Figure 2. Following the evaluation of overall discrimination, classification performance metrics were calculated for each model based on the optimal cutoff (Table 3). The negative predictive values of all three models exceeded 90%. In the Lu Qiufang model, the positive predictive value (48.4%), specificity (84.9%), accuracy (82.1%), and Youden index (0.536) were the highest among the three models. In the Xi Pan model, the corresponding values were 39.3%, 79.3%, 76.9%, and 0.443, while in the Shen Xiaofang model, they were 28.0%, 64.7%, 65.0%, and 0.315.

**Table 3:** Discrimination and classification performance of the three DVT risk prediction models.

Model	AUC (95% CI)	Sen (%)	Spe (%)	Acc (%)	PPV (%)	NPV (%)	YI
Shen Xiaofang model	0.699 (0.660–0.739)	66.80	64.70	65.00	28.00	90.40	0.32
Lu Qiufang model	0.804 (0.768–0.840)	68.70	84.90	82.10	48.40	92.90	0.54
Xi Pan model	0.753 (0.714–0.792)	65.00	79.30	76.90	39.30	91.70	0.44

AUC, area under the receiver operating characteristic curve; Sen, sensitivity; Spe, specificity; Acc, accuracy; PPV, positive predictive value; NPV, negative predictive value; YI, Youden index.

### 3.4 Model calibration

**Figure 3:**
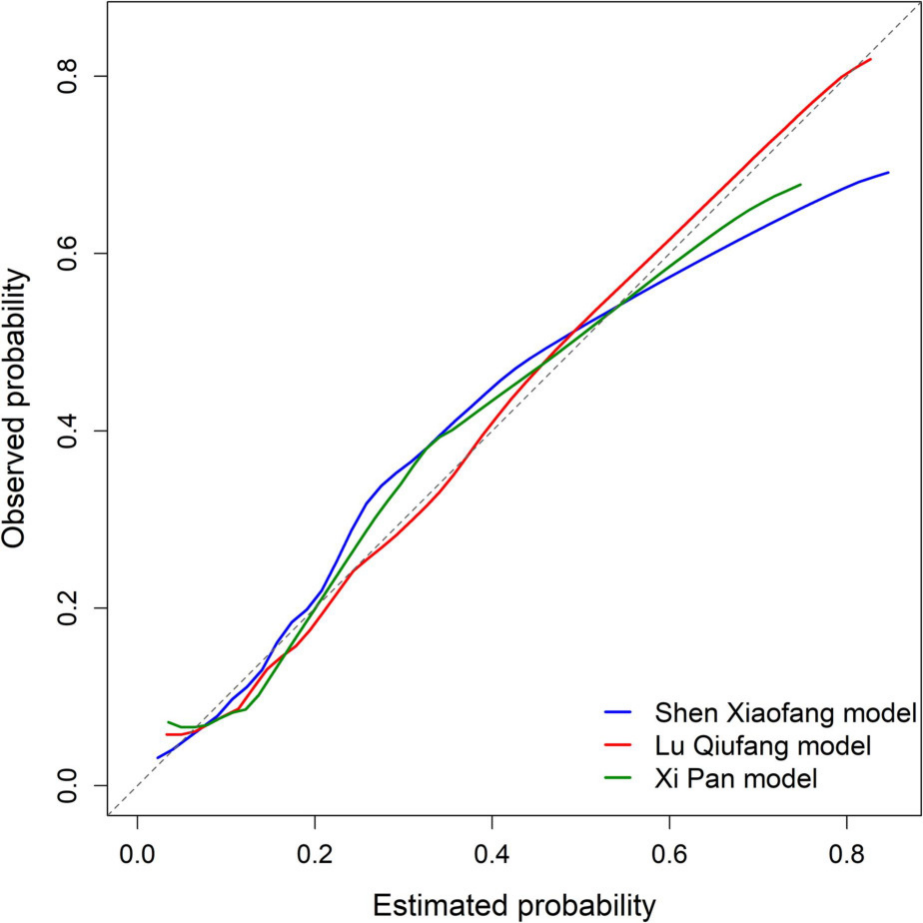
Comparison of Bootstrap calibration curves among three DVT risk prediction models.

To evaluate the agreement between predicted probabilities and actual incidence, calibration curves were constructed using the bootstrap resampling method (*b* = 1,000) (Figure 3). Under ideal calibration, the prediction curve should align closely with the 45-degree reference line. The calibration curves of all three models were generally close to the reference line across different probability intervals, without substantial systematic deviation. The Lu Qiufang model showed the best fit, with its calibration curve remaining close to the reference line in most probability intervals. In contrast, the Xi Pan and Shen Xiaofang models had calibration curves below the reference line in the high-probability range, indicating that predicted risks were higher than the observed incidences. We further assessed the overall prediction error using the Brier score, which reflects the discrepancy between predicted probabilities and actual outcomes, with lower values indicating smaller errors. The Brier score was 0.154 for the Lu Qiufang model, 0.182 for the Shen Xiaofang model, and 0.245 for the Xi Pan model.

### 3.5 Clinical utility of the model

**Figure 4:**
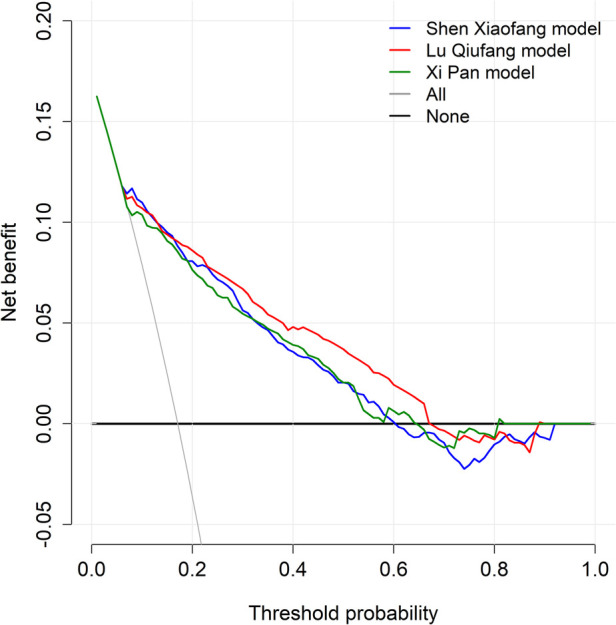
Comparison of DCA curves among three DVT risk prediction models.

To further evaluate the clinical applicability of the three prediction models, DCA was performed to quantify the net benefit across different threshold probabilities (Figure 4). The “None” line represents the reference net benefit of withholding intervention from all patients, while the “All” line indicates the theoretical reference value if all patients were treated. The decision curves of all three models were mostly above the “None” line within the threshold probability range of approximately 0.05–0.80, indicating positive net benefit in this interval. The Lu Qiufang model consistently showed the highest net benefit between 0.20 and 0.70, with its curve remaining above those of the other two models. The Xi Pan model demonstrated slightly higher net benefit than the Shen Xiaofang model across most thresholds, with their curves remaining close. After approximately 0.58, the Shen Xiaofang model fell below the “None” line, and after 0.64, the Xi Pan model gradually approached the “None” line. The Lu Qiufang model began to decline after around 0.80.

## 4 Discussion

### 4.1 Analysis of differences in variable composition among the three models

The Shen Xiaofang model incorporated the Padua score as an independent predictor, along with age, diabetes mellitus, dyslipidemia, D-dimer, and limb muscle strength. Some of these variables overlap with components already included in the Padua score, resulting in redundant information within the model. This redundancy may lead to collinearity and increase the risk of overfitting, thereby undermining the model's generalizability in external validation (21). In addition, overlapping variables increase the structural complexity of the model, raising the burden of clinical assessment and implementation costs, which reduces its practicality and limits its applicability in clinical promotion (21, 22).

In contrast, the Lu Qiufang model adopts a more concise variable selection strategy, including only four indicators—age, state of consciousness, degree of hemiplegia, and use of dehydrating drugs. These variables are clearly defined and easy to collect rapidly, allowing assessment to be completed even without auxiliary examinations. This makes the model particularly suitable for settings with limited resources or where rapid evaluation is required. However, two of its core variables—state of consciousness and degree of hemiplegia—are based on clinical observation and may be subject to variability among different evaluators. Such variability may compromise data consistency and model stability. To enhance reproducibility and external applicability, further standardization of these assessments in clinical practice is needed.

The Xi Pan model primarily uses predictors derived from medical records and laboratory test results. This standardized approach reduces the influence of subjective factors, thereby ensuring data quality and model stability. However, its reliance on laboratory indicators such as albumin and D-dimer may not fully meet the need for rapid risk prediction in urgent clinical situations (23).

Furthermore, the three models differ in their treatment of variables such as age, limb muscle strength, and D-dimer levels. The Shen Xiaofang model treats these as continuous variables to preserve maximal information, whereas the Lu Qiufang and Xi Pan models categorize them to improve clinical usability and interpretability. Previous studies have suggested that, without a clear rationale, converting continuous variables into categorical ones may cause information loss and reduce predictive performance (24, 25). Therefore, the choice of variable handling methods should strike a balance between clinical convenience and predictive accuracy (26).

### 4.2 Comparison of discrimination and classification performance among the three models

AUC is commonly used to evaluate the discriminatory ability of prediction models. An AUC of 0.70–0.80 is generally considered moderate, 0.80–0.90 good, and greater than 0.90 excellent, whereas values below 0.70 indicate poor discrimination (27). In the external validation of this study, the Lu Qiufang model achieved an AUC of 0.804, indicating good discriminatory ability. For classification performance, its positive predictive value was 48.4% (moderate), specificity 84.9% (good), accuracy 82.1% (good), Youden index 0.536 (good), and the negative predictive value exceeded 90% (excellent). Overall, this model performed particularly well in ruling out low-risk patients, although its precision in identifying high-risk patients still requires improvement.

The Xi Pan model achieved an AUC of 0.753, which falls within the range of moderate discrimination. For classification performance, its positive predictive value was 39.3% (moderate), specificity 79.3% (upper-moderate), accuracy 76.9% (upper-moderate), Youden index 0.443 (lower-good), and the negative predictive value exceeded 90% (excellent). Overall, the model showed only average classification performance. It may be suitable for preliminary risk screening, but caution is needed to avoid misclassification.

The Shen Xiaofang model had an AUC of 0.699, close to the threshold for poor discrimination. For classification performance, its positive predictive value was only 28.0% (poor), specificity 72.2% (lower-moderate), accuracy 65.0% (poor), Youden index 0.315 (lower-moderate), and the negative predictive value exceeded 90% (excellent). Although the model demonstrated good negative predictive ability, its overall classification performance was clearly inadequate, particularly in identifying high-risk patients. Its clinical utility is therefore limited, and further studies are needed to optimize its structure and variable selection.

### 4.3 Calibration consistency and clinical benefit evaluation

Calibration reflects the agreement between predicted probabilities and actual outcomes (28). In this study, calibration was evaluated using calibration curves and Brier scores, while DCA was applied to further assess the net clinical benefit of each model across different threshold probabilities (21). From the perspective of calibration consistency, the Lu Qiufang model showed calibration curves that closely followed the ideal reference line across different probability intervals, indicating good agreement between predicted probabilities and actual outcomes. Its Brier score was 0.154, the lowest among the three models, further demonstrating its advantage in controlling overall prediction error. The DCA curve also showed that the Lu Qiufang model maintained consistently higher net benefit within the threshold range of approximately 0.20–0.70, suggesting strong potential for clinical decision support in this interval. This may provide clinicians with a more reliable basis for risk assessment, thereby optimizing intervention strategies and improving the precision and effectiveness of patient management.

By contrast, the calibration curve of the Shen Xiaofang model was clearly below the ideal reference line in the medium- to high-risk intervals, indicating a systematic tendency to overestimate risk in this range, with predicted probabilities generally higher than the observed incidence. Although its Brier score was 0.182, still within an acceptable range and not reflecting substantial overall prediction error, the bias in specific intervals may interfere with clinical risk assessment. The DCA results also reflected this limitation: when the threshold probability exceeded approximately 0.58, its net benefit curve fell below the “None” line, suggesting that interventions based on the model's predictions at this threshold could lead to unnecessary treatment for patients who are actually at low risk, thereby reducing the overall clinical net benefit.

The Xi Pan model showed the opposite trend in calibration. Its calibration curve was clearly above the reference line in the low-risk range, indicating systematic underestimation, with predicted probabilities lower than the actual risk for some high-risk patients, which may lead to insufficient clinical recognition. Its Brier score was 0.245, the highest among the three models, further indicating relatively greater overall prediction error. The DCA curve also showed that its net benefit was slightly higher than that of the Shen Xiaofang model across most thresholds but consistently lower than that of the Lu Qiufang model. Thus, its clinical applicability is relatively limited and should be judged cautiously in relation to specific clinical contexts.

In this study, all three externally validated models were presented as nomograms. Although this provides a certain degree of risk visualization, reliance on manual calculation is insufficient to meet the demands of rapid decision-making and large-scale application in real clinical settings. At present, predictive models in clinical practice commonly face the dilemma of being “predictable but difficult to apply,” mainly due to limited interpretability, cumbersome operational processes, and poor alignment with clinical workflows. The value of a prediction model should not lie solely in the output of risk probabilities, but more importantly in supporting actual clinical judgment and decision-making. If a model cannot be integrated into the clinical decision-making process of healthcare providers, its clinical translation value will remain limited, even with strong predictive performance. As an essential tool for achieving a closed loop of clinical decision-making, prediction models should be application-oriented and truly promote the deep integration of their outputs with clinical workflows.

## 5 Conclusions

This study externally validated three DVT risk prediction models using clinical data from patients with acute stroke and comprehensively evaluated their discrimination, calibration, and clinical decision benefit. The results showed that, compared with the other two models, the Lu Qiufang model demonstrated relatively superior performance across multiple indicators, with higher overall stability within this cohort, providing supportive evidence for model selection in similar clinical settings.

This study was a single-center validation conducted in a tertiary hospital, which may limit the representativeness and transportability of the findings. Future research should include multicenter and cross-regional external validation studies to further assess model robustness and generalizability across diverse clinical environments. In addition, embedding validated models into hospital electronic medical record systems or clinical decision support systems may facilitate automated risk assessment and improve practical implementation efficiency.

## Data Availability

The raw data supporting the conclusions of this article will be made available by the authors, without undue reservation.
