# Supplementary material for: External validation of three risk prediction models for deep vein thrombosis in patients with acute stroke: a single-center cohort study
Source: Front Cardiovasc Med. 2026 Apr 10;13:1753784. doi: 10.3389/fcvm.2026.1753784 (PMC13106147; doi:10.3389/fcvm.2026.1753784)
Supplement: Supplementary file 1 [file Datasheet1.pdf]

Supplementary Material

1 Supplementary Figures and Tables

1.1 Supplementary Figures

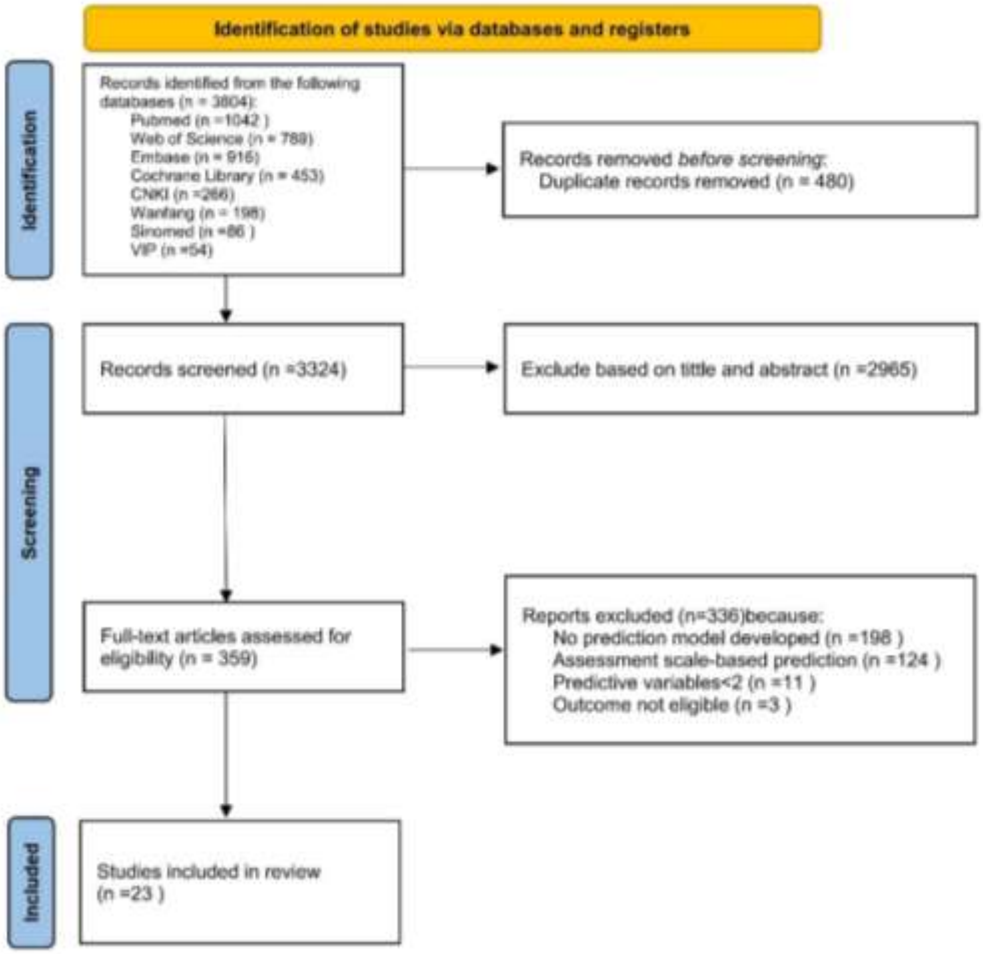

Figure S1. PRISMA 2020 Flow Diagram of Study Selection

1.2 Supplementary Tables

Table S1. Detailed PubMed Search Strategy

No.	Entry terms
1	"Stroke"[Mesh] OR ("Stroke, Acute"[Title/Abstract] OR "Acute Stroke"[Title/Abstract] OR "Cerebrovascular Accident"[Title/Abstract] OR "Cerebral Stroke"[Title/Abstract] OR "Cerebrovascular Apoplexy"[Title/Abstract] OR "Brain Vascular Accident"[Title/Abstract] OR "CVA"[Title/Abstract] OR "Acute Cerebrovascular Accident"[Title/Abstract])

2	"Venous Thromboembolism"[Mesh] OR "Venous Thrombosis"[Mesh] OR "Deep Vein Thrombosis"[Title/Abstract] OR "Deep Venous Thrombosis"[Title/Abstract] OR "Phlebothrombosis"[Title/Abstract]
3	"Risk Factors"[Mesh] OR "Risk Assessment"[Mesh] OR "Nomograms"[Mesh] OR "Logistic Models"[Mesh] OR "Models, Statistical"[Mesh] OR Predict*[Title/Abstract] OR Model*[Title/Abstract] OR "Risk Score"[Title/Abstract] OR "Risk Prediction Model"[Title/Abstract] OR "Risk Stratification"[Title/Abstract] OR Nomogram*[Title/Abstract] OR "Machine Learning"[Title/Abstract]
4	1 AND 2 AND 3 (n = 3,804 before deduplication)

Table S2. Inclusion and Exclusion Criteria for the Systematic Review

Category	Criteria
Inclusion Criteria	1. Study population: Patients with imaging-confirmed stroke.<br>2. Study design: Case-control, cohort, or cross-sectional studies.<br>3. Study content: Development and/or validation of multivariable (≥2 predictors) risk prediction models for DVT in patients with stroke.<br>4. Outcome: Deep vein thrombosis (DVT).<br>5. Language: English or Chinese.
Exclusion Criteria	1. Reviews, case reports, or other non-original publications.<br>2. Duplicate publications.<br>3. Studies without accessible full text or with incomplete key data.

Table S3. Characteristics of DVT Prediction Models in Stroke

Author (Year)	Population	Outcome	Predictors	Discrimination (AUC)	Model Presentation
Li Zhiheng (2024)	Ischemic stroke	DVT	Age, dehydrating therapy, bed rest duration, D-dimer	0.839	Regression formula
Zhang Yan (2024)	Hemorrhagic stroke	DVT	Mechanical ventilation, hematoma volume, femoral catheterization, coagulation time	0.813	Nomogram

Zhang Shasha (2024)	Hemorrhagic stroke	DVT	Age, smoking, hypertension, heart disease, hematoma volume, surgery duration	0.938	Regression formula
Ding Minghui (2024)	Hemorrhagic stroke	DVT	TAT complex, thrombomodulin, PAP complex	0.830	Nomogram
Hang Dai (2024)	Ischemic stroke	DVT	Age, diabetes, NIHSS, bed rest, platelets, fibrinogen, D-dimer	0.850	Nomogram
Ma Yaying (2024)	Ischemic stroke	DVT	Dysphagia, dehydrating therapy, D-dimer	0.951	Not clearly specified
Wu Li (2024)	Hemorrhagic stroke	DVT	Bed rest, infection, hematocrit, muscle strength, transfusion, D-dimer	0.950	Not clearly specified
Lingling Liu (2024)	stroke	DVT	Age, stroke type, diabetes, mobility, D-dimer, etc.	0.740	Web calculator
Hai Xu (2024)	Ischemic stroke	DVT	Age, diabetes, NIHSS, D-dimer, fibrinogen	0.829	Nomogram
Xiao Yi (2023)	Ischemic stroke	DVT	AF, pneumonia, D-dimer, NIHSS, muscle strength, age	0.895	Nomogram
Shen Xiaofang (2023)	stroke	DVT	Age, diabetes, dyslipidemia, Padua score, D-dimer, muscle strength	0.890	Nomogram
Wang Wenhui (2023)	Hemorrhagic stroke	DVT	Age, hypertension, bed rest, dehydrating therapy, homocysteine	Not clearly reported	Regression formula

Yan Feifan (2022)	Hemorrhagic stroke	DVT	Age, sex, consciousness, muscle strength, season, D-dimer	0.766	Nomogram
Lu Qiufang (2022)	stroke	DVT	Dehydrating therapy, hemiplegia, consciousness, age	0.850	Nomogram
Haoran Cheng (2021)	Ischemic stroke	DVT	Age, sex, paralysis, malignancy, pneumonia, AF	0.820	Nomogram
Xi Pan (2021)	stroke	DVT	Age, sex, stroke type, malignancy, muscle strength, albumin, D-dimer	0.756	Nomogram
Miao Yun (2021)	Ischemic stroke	DVT	NIHSS, bed rest, D-dimer, lipids	0.876	Regression formula
Chen Huijiao (2021)	Hemorrhagic stroke	DVT	Age, sedatives, hypertension, D-dimer	0.912	Regression formula
Huang Jie (2021)	Ischemic stroke	DVT	Age, infarct location, muscle strength, fibrinogen, platelets	0.769	Nomogram
Hu Xilian (2020)	Ischemic stroke	DVT	Diabetes, AF, NIHSS, D-dimer, creatinine	0.877	Nomogram
Ye Xiaofeng (2020)	Hemorrhagic stroke	DVT	Age, surgery type, smoking, catheterization	0.710	Nomogram
Xu Yan (2020)	Hemorrhagic stroke	DVT	Hematoma volume, bed rest, homocysteine, D-dimer	0.831	Nomogram

Liping Liu (2014)	stroke	DVT	Age, sex, BMI, cancer, stroke type, NIHSS	0.700	Nomogram

Table S4. Risk of Bias and Applicability Assessment of Included Prediction Models

Author (Year)	ROB				Applicability			Overall	
	**Participants**	**Predictors**	**Outcome**	**Analysis**	**Participants**	**Predictors**	**Outcome**	**ROB**	**Applicability**
Li Zhiheng (2024)	-	+	+	-	-	+	+	-	-
Zhang Yan (2024)	-	+	+	-	-	+	+	-	-
Zhang Shasha (2024)	-	+	+	-	-	+	+	-	-
Ding Minghui (2024)	-	+	+	-	-	+	+	-	-
Hang Dai (2024)	-	+	+	-	-	+	+	-	-
Ma Yaying (2024)	-	+	+	-	-	+	+	-	-
Wu Li (2024)	-	+	+	-	-	+	+	-	-
Lingling Liu 2024	-	+	+	-	+	+	+	-	+
Hai Xu	-	+	+	-	-	+	+	-	-

2024									
Xiao Yi (2023)	+	+	+	-	-	+	+	-	-
Shen Xiaofang (2023)	-	+	+	-	+	+	+	-	+
Wang Wenhui (2023)	-	+	?	-	-	+	+	-	-
Yan Feifan (2022)	-	+	+	-	-	+	+	-	-
Lu Qiufang (2022)	+	+	+	-	+	+	+	-	+
Haoran Cheng 2021	-	+	+	-	-	+	+	-	-
Xi Pan 2021	+	+	+	-	+	+	+	-	+
Miao Yun (2021)	-	+	+	-	-	+	+	-	-
Chen Huijiao (2021)	?	+	+	-	-	+	+	-	-
Huang Jie (2021)	?	+	+	-	-	+	+	-	-
Hu Xilian (2020)	-	+	+	-	-	-	+	-	-

Ye Xiaofeng (2020)	-	+	?	-	-	+	+	-	-
Xu Yan (2020)	-	+	+	-	-	+	+	-	-
Liping Liu 2014	+	?	+	-	+	+	+	-	+

Note:PROBAST, Prediction model Risk Of Bias Assessment Tool; ROB, risk of bias.
+ indicates low ROB/low concern regarding applicability; - indicates high ROB/high concern regarding application; ? indicates unclear ROB/unclear concern regarding applicability.

Table S5. Variable coding and regression coefficients of the prediction models

Model	Variable	Classification	Coding	β
Shen Xiaofang	Age		Original value	0.347
	Diabetes Mellitus	No / Yes	0 / 1	0.543
	Dyslipidemia	No / Yes	0 / 1	0.614
	Padua Score		Original value	1.215
	D-dimer		Original value	0.456
	Muscle Strength		Original value	−0.542
Lu Qiufang	Age	≤60; 61–74; ≥75	0 / 1 / 2	0.643
	State of Consciousness	Clear; Lethargic; Somnolent; Comatose	0–3	0.532
	Degree of Hemiplegia	Normal; Mild; Incomplete; Complete	0–3	0.663
	Use of Dehydrating Agents	No / Yes	0 / 1	1.173
Xi Pan	Age	<65; ≥65 years	0 / 1	0.343
	Sex	Male; Female	0 / 1	0.595
	Stroke Type	Ischemic; Hemorrhagic	0 / 1	0.894
	Malignant Tumor	No / Yes	0 / 1	0.856
	D-dimer	≤0.5; >0.5	0 / 1	1.263
	Lower Limb Muscle Strength	≥3; <3	0 / 1	0.632
	Serum Albumin	<40; 40–55; >55	0 / 1 / 2	0.469
